# Perceptions of lived in experiences of healthcare workers during COVID-19 Pandemic in a tertiary Care hospital: A perspective from Pakistan

**DOI:** 10.12669/pjms.40.8.9010

**Published:** 2024-09

**Authors:** Bushra Ameer Saeed Awan, Shamaila Mohsin, Syed Fawad Mashhadi, Mohi Ud Din

**Affiliations:** 1 Bushra Ameer Saeed Awan, MBBS, MPhil Demonstrator, Department of Community Medicine/Public Health, Army Medical College/ National University of Medical Sciences, Rawalpindi, Pakistan; 2 Shamaila Mohsin, MBBS, MPH, MPhil, Ph.D Associate. Professor, Department of Community Medicine/Public Health, Army Medical College/ National University of Medical Sciences, Rawalpindi, Pakistan; 3 Syed Fawad Mashhadi, MBBS, MPH,MCPS, MPhil, Ph.D Professor/ Head of Department, Department of Community Medicine/Public Health, Army Medical College/ National University of Medical Sciences, Rawalpindi, Pakistan; 4 Mohi Ud Din, MBBS, MPhil Assistant professor, Community Medicine Dept, Aziz Fatimah Medical and Dental College, Faisalabad

**Keywords:** COVID-19, Experiences, Healthcare Workers, Perceptions, Pandemic, Qualitative

## Abstract

**Objective:**

This study aimed to explore the perceptions of frontline Healthcare Workers (HCWs) towards managing COVID-19 in Tertiary care hospital in Pakistan.

**Method:**

This qualitative exploratory study was conducted from January to May 2021 at a Tertiary care hospital designated for COVID-19 patients in Rawalpindi Pakistan. Semi-structured in-depth interviews were conducted from twenty six HCWs. To acquire a sample that was diverse in terms of professional capacity, degree of experience and exposure, purposive sampling technique was used. After thematic analysis, themes were generated by identifying patterns among codes.

**Results:**

Five themes emerged. All HCWs experienced significant amount of negative emotions including fear, uncertainty, imposing social distancing and workload. There were also positive experiences such as rewarding, call of duty and professional growth. HCWs experiences related to personal protective equipment (PPE) were communication issues, physical and dermatological issues, reuse and working confidently while wearing PPE. HCWs were buoyed by cooperation and facilitation, trainings and health education from administration. Coping mechanisms were used such as seeking team support, adjusting cognition to deal with reality and resorting to religion.

**Conclusion:**

Negative emotions predominated in the beginning and positive emotions emerged gradually. PPE and administrative support played significant role. HCWs’ mental health was maintained in part by their self-coping styles. The findings of this study can be employed to inform and enhance future pandemic response initiatives.

## INTRODUCTION

COVID-19 pandemic has been a major test for strengths and vulnerabilities of health-care systems around the world. Each succeeding wave of the COVID-19 was more lethal than the previous one leading to exacerbation of previous stressful events.^1^ The possible impact of COVID-19 on local, national and international levels on healthcare systems was significant. The continuous rise in the number of COVID-19 infections corresponded with a simultaneous increase in the incidence of COVID-19 cases among Healthcare Workers (HCWs).^2^ COVID-19 affected HCWs and ultimately impacted the healthcare system in handling the crisis.

Low middle income countries (LMIC) with less developed health systems were likely to experience more difficulties in controlling COVID-19 pandemic compared to the High-Income Countries (HIC).^3^ Evidence from HIC identified the highly infectious nature of the virus, fear of transmitting infection to family, social isolation, stigma, lack of communication with colleagues due to strict isolation measures, confidence in organizational support as main factors influencing the behavior of HCWs during the pandemic.^4^ Studies from LMIC identified inadequate resources, fear of getting infected, poor staff welfare, inadequate logistics, misinformation, difficulty in implementing social distancing, vulnerability to contract infection, shortage of Personal Protective Equipment (PPE) and reduced contact with family and friends were challenges faced by HCWs.^5^

The pandemic stressed the entire healthcare system of Pakistan and outpaced the capacity of hospitals to meet the increasing demand.^6^ In order to guide successful workplace and national responses during future healthcare emergencies, qualitative studies from previous pandemics stressed the importance of documenting the in-depth perspectives of frontline HCWs.^7^ The aim of this study was to find out the experiences of HCWs rendering services during COVID-19 pandemic.

## METHOD

This qualitative study was conducted from January to May 2021 at a Tertiary care hospital designated for COVID-19 patients in Rawalpindi Pakistan.Medical Specialists, resident doctors, house officers, nurses and paramedics who provided direct care for patients with confirmed COVID-19 infection from both clinical and administrative side were included in the study. HCWs who did not have potential direct/ indirect interaction with COVID-19 patients, had chronic, terminal, psychiatric illnesses and unwilling to participate in the study were excluded.

### Ethical Approval:

The study was approved from the Ethical Review Committee of Army Medical College, National University of Medical Sciences (ERC/ID/87) dated Nov 30, 2020.

**Fig.1:**
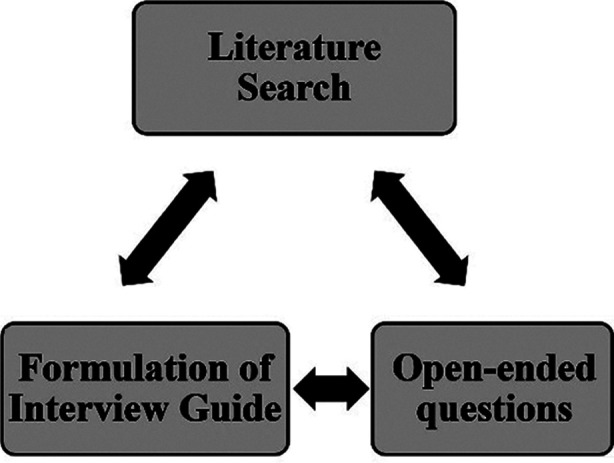
Formulation of Interview Guide.

To acquire a sample that was diverse in terms of professional capacity, degree of experience, and exposure, purposive sampling technique was used. Data gathering and analysis was concurrent with each other to determine when saturation was achieved, when no new codes and themes emerged and all identified themes were sufficiently supported by the data collected. After extensive literature review, semi structured interview guide was designed based on recommendations from a qualitative semi structured interview guide.^8^ Process of formulation of interview guide is shown in Fig.1.

The main interview questions posed to the participants were the experiences of taking care of patients with COVID-19, difference between providing care during the pandemic and routine duties, feelings in the initial phase, specific challenges encountered and how did they respond, support from administration, experience with PPE and training regarding PPE. By using designation, age and gender instead of names and removing identifying information from the transcripts confidentiality, anonymity and privacy was assured. Throughout this study, the Standards for Reporting Qualitative Research guidelines were followed.^9^

**Fig.2:**
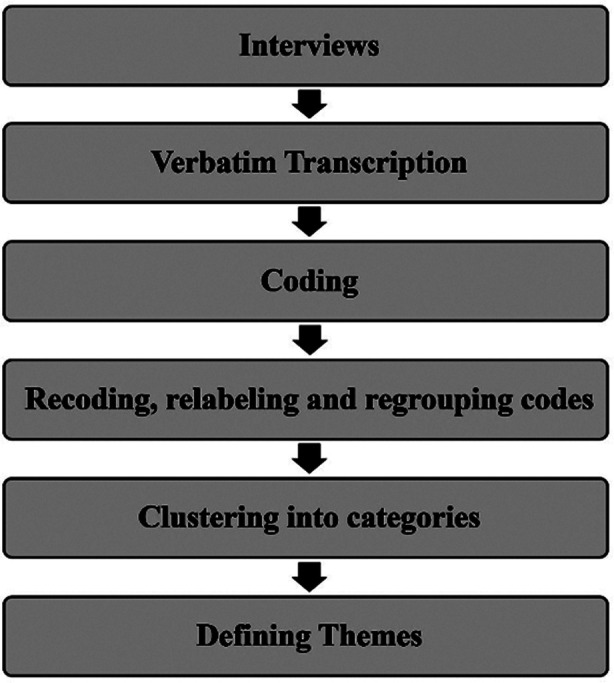
Process of Thematic Analysis.

Thematic analysis which is one of the most common forms of qualitative data analysis was applied in this study. To ensure the accuracy of the information, the recording were transcribed into text within 24 hours after the interviews. During the thematic analysis process, a thorough overview of all the data was first completed by reading and rereading of the transcripts. Process of thematic analysis is illustrated in Fig.2.

## RESULTS

In total, twenty six in depth interviews (IDIs) were conducted after taking informed consent as thematic saturation had been reached. Study sample consisted of medical specialists n=4(15%), resident doctors n=5(19%), house officers n=6(23%), registered nurses n=8(31%) and paramedics n=3(12%).Age of participants ranged from 21 years to 55 years with average 33.2± 9.39 years. Majority n=17(65%) were females and n=9 (35%) males. A thorough review of themes was done to guarantee their utility and accuracy in reflecting the data, which involved actions such as segmenting, consolidating, eliminating, or creating new themes as needed. Five theme and seventeen subtheme categories emerged after through the identification of patterns among codes, while simultaneously conducting a search for data pertinent to each theme shown in Table-I.

**Table-I:** Experiences of Healthcare Workers in COVID-19.

Theme	Sub theme	Quote
Negative Perceptions	Fear	“Psychologically for yourself you are not that anxious, but you are afraid that you may give this disease to your family members that was a very big fear” (Resident, M, Age 32)
	Uncertainty	“Since it was a new disease and we didn’t not know anything about that we did not know when it would hit and how hard it would hit” (Medical Specialist, F, Age 55)
	Social distancing	“As social interaction amongst colleague was decreased, everyone was afraid of contracting infection, avoided each other and kept a safe distance” (Nurse, F, Age 42)
	Workload	“Workload increased a lot, with respect to resources and manpower, as many doctors and nurses got infected or on quarantine” (House Officer, F, Age 26)
Positive Perceptions	Rewarding	“It is very rewarding because those patients who came to us their prognosis was good and deterioration rate was less, so we got a very positive feeling” (Resident, M, Age 29)
	Sense of duty	“The part of my profession you can’t run away from it, you have to be there and provide your services when you are needed the most” (House Officer, M, Age 24)
	Growth and Learning	“Professionally we learned a lot and less resources are , by managing them appropriately and plan well you will always end up with success” (Medical specialist, F, Age 55)
Experiences with Personal Protective Equipment (PPE)	Communication issues	“It was very difficult communicating with patient with double mask and our voice couldn’t reach patients” (Medical Specialist, F, Age 37)
	Physical and Dermatological issues	. “It was physically exhausting wearing PPE, after coming back from hospital I had rashes all over body because of wearing PPE for so long and allergy to latex in gloves” (House Officer, F, Age 24)
	Reuse of PPE	“These washable coveralls, didn’t appear trustworthy to me as are sanitized after every use” (Nurse, F, Age 34)
	Work Confidently with PPE	“Once we realized that PPE is working and most of us who wore proper PPE didn’t contract COVID-19 they were like any other patients” (House Officer, M, Age 25)
Experiences with Admin-istration	Administrative support	“Administration addressed all of our genuine demands, nothing that we tell them about our issue, and it was not addressed” (Resident, M, Age 32)
	Trainings	“We were given training with regard to use of PPE and hand hygiene. It was hands on training given by medical officer in charge” (Paramedic, M, Age 43)
	Health Education	“There were info graphics and pan flex regarding proper donning and doffing and hand hygiene in each donning and doffing area” (House Officer, F, Age 23)
Coping strategies	Team spirit	“In the end we realized okay we are all together in this, so you learn to do it as a teamwork” (Resident, M, Age 32)
	Adjusting cognition	“I just went with the flow because when you know there is no other way. You had to bear with it because this is who we are” (Resident, M, Age 32)
	Resorting to Religion	“I used to offer prayers, pray for this menace to end soon and pray for my children and family” (Medical specialist, F, Age 40)

## DISCUSSION

This study explored the lived in experiences of HCWs working in COVID-19 using thematic analysis approach. Findings of this study are summarized as negative experiences, positive perceptions, experience with PPE, administrative experiences and coping strategies.

HCWs were overwhelmed with fear of catching infection and taking the virus home. These findings are congruent with a study conducted in Mexico.^10^ Frequent duties and long working hours resulted in increased workload which is consistent with study conducted on HCWs in the United States.^11^ Things were not streamlined in the early stages of the pandemic therefore HCWs were faced with uncertainty. These findings corroborate with a study conducted on physicians, nurses and other HCWs in Saudi Arabia.^12^ HCWs also reported the negative impact of social distancing on their lives, similar findings were reported in a study conducted in China.^13^

Along with negative emotions, and HCWs had positive experiences as well. They were proud of themselves for having the courage and potential to overcome challenges and found value in their COVID-19 experience in line with a scoping review conducted on wellness of HCWs during COVID-19 pandemic.^14^ Physical discomfort 15 due to prolonged PPE use, skin breaches and irritation, caused HCWs to take time off.^16^ Many described their dissatisfaction with reuse of PPE that could be source of infection similar to a systematic review conducted by Chughtai et al.^17^ Wearing PPE gave HCWs a sense of protection, which led to better caregiving for COVID-19 patients which is in conformity with study conducted by Gan et al.^18^

The appreciation and regard of the study participants for the support and supportive working environments which motivated staff to render duties in challenging circumstances of delivering care in the context of a pandemic in contrast to study conducted by Bropy JT et al., in Canada.^19^ HCWs received training regarding the use of PPE and IPC procedures which made them feel confident and prepared to deliver care which corroborates with study conducted in Singapore.^20^

HCWs also counted on peer support and self-adjustment skills to cope with the psychological issues in agreement with a systematic review and meta-analysis conducted by Sibeoni J. et al^21^ HCWs regarded religion as an important support in maintaining psychological well-being in line with study conducted in Malaysia.^22^ The findings of this study point to a progression from initial confusion and anxiety at the start to a focus on preparation, and then reaping the consequences of that preparedness and professionalism Evidence from Austria supported similar findings.^23^ The majority of HCWs reported religiously following preventive measures while at work and after duty hours and social isolation. A qualitative review conducted by Houghton C et al documented similar results.^24^

Although few studies looked into HCWs mental health issues and factors contributing to stress and anxiety, but all those studies were conducted online.^25^ In this study, interviews were conducted in person and nonverbal cues such as facial expressions, eye contact and body movements, which is the strenght of this study.

### Limitations of the Study:

Although this study provided some interesting insights it was not without limitations. This was a study spanned over period of few months. Long-term research topic experience will be a worthwhile path to pursue in the future.

## CONCLUSION

This research offered a detailed and in-depth understanding of HCWs’ psychological experiences during COVID-19 pandemic. Negative emotions predominated in the beginning, positive emotions emerged gradually. Proper PPE and provision of administrative support was highlighted as a necessary component. HCWs’ mental health was maintained in part by their self-coping styles and psychological development. The findings of this study can be employed to inform and enhance future pandemic response initiatives.

### Authors’ Contribution:

**BASA:** Conception, design, data collection, analysis, manuscript draft, final approval of manuscript, critical revision.

**SM:** Conception, design, review, final approval of manuscript, accountability.

**SFM:** Final approval of manuscript, review, accountability.

**MOD:** Review, Responsible for integrity of research..

## References

[1] Chokshi DA, Katz MH (2020). Emerging lessons from covid-19 response in new york city. JAMA.

[2] Driggin E, Madhavan MV, Bikdeli B, Chuich T, Laracy J, Biondi-Zoccai G (2020). Cardiovascular considerations for patients, health care workers, and health systems during the COVID-19 pandemic. J Am Coll Cardiol.

[3] Peters A, Vetter P, Guitart C, Lotfinejad N, Pittet D (2020). Understanding the emerging coronavirus:what it means for health security and infection prevention. J Hosp Infect.

[4] Vindrola-Padros C, Andrews L, Dowrick A, Djellouli N, Fillmore H, Bautista Gonzalez E (2020). Perceptions and experiences of healthcare workers during the COVID-19 pandemic in the UK. BMJ Open.

[5] Okediran JO, Ilesanmi OS, Fetuga AA, Onoh I, Afolabi AA, Ogunbode O (2020). The experiences of healthcare workers during the COVID-19 crisis in Lagos, Nigeria:A qualitative study. Germs.

[6] Feroz AS, Pradhan NA, Hussain Ahmed Z, Shah MM, Asad N, Saleem S (2021). Perceptions and experiences of healthcare providers during COVID-19 pandemic in Karachi, Pakistan:an exploratory qualitative study. BMJ Open.

[7] Kim Y (2018). Nurses'experiences of care for patients with Middle East respiratory syndrome-coronavirus in South Korea. Am J Infect Control.

[8] Kallio H, Pietilä AM, Johnson M, Kangasniemi M (2016). Systematic methodological review:developing a framework for a qualitative semi-structured interview guide. J Adv Nurs.

[9] O'Brien BC, Harris IB, Beckman TJ, Reed DA, Cook DA (2014). Standards for reporting qualitative research:a synthesis of recommendations. Acad Med.

[10] García-Reyna B, Castillo-García GD, Barbosa-Camacho FJ, Cervantes-Cardona GA, Cervantes-Pérez E, Torres-Mendoza BM (2022). Fear of COVID-19 Scale for hospital staff in regional hospitals in Mexico:A brief report. Int J Ment Health Addict.

[11] Arnetz JE, Goetz CM, Arnetz BB, Arble E (2020). Nurse Reports of Stressful Situations during the COVID-19 Pandemic:Qualitative Analysis of Survey Responses. Int J Environ Res Public Health.

[12] Al-Mansour K, Alfuzan A, Alsarheed D, Alenezi M, Abogazalah F (2021). Work-Related Challenges among Primary Health Centers Workers during COVID-19 in Saudi Arabia. Int J Environ Res Public Health.

[13] Zhang H, Tang L, Ye Z, Zou P, Shao J, Wu M (2020). The role of social support and emotional exhaustion in the association between work-family conflict and anxiety symptoms among female medical staff:a moderated mediation model. BMC Psychiatry.

[14] Shreffler J, Petrey J, Huecker M (2020). The Impact of COVID-19 on Healthcare Worker Wellness:A Scoping Review. West J Emerg Med.

[15] Kim Y (2018). Nurses'experiences of care for patients with Middle East respiratory syndrome-coronavirus in South Korea. Am J Infect Control.

[16] Singh M, Pawar M, Bothra A, Maheshwari A, Dubey V, Tiwari A (2020). Personal protective equipment induced facial dermatoses in healthcare workers managing Coronavirus disease 2019. J Eur Acad Dermatol Venereol.

[17] Chughtai AA, Khan W (2020). Use of personal protective equipment to protect against respiratory infections in Pakistan:A systematic review. J Infect Public Health.

[18] Gan WH, Lim JW, Koh D (2020). Preventing Intra-hospital Infection and Transmission of Coronavirus Disease 2019 in Health-care Workers. Saf Health Work.

[19] Brophy JT, Keith MM, Hurley M, McArthur JE (2021). Sacrificed:Ontario Healthcare Workers in the Time of COVID-19. New Solut.

[20] Wong JEL, Leo YS, Tan CC (2020). COVID-19 in Singapore-Current Experience:Critical Global Issues That Require Attention and Action. JAMA.

[21] Sibeoni J, Bellon-Champel L, Mousty A, Manolios E, Verneuil L, Revah-Levy A (2019). Physicians'Perspectives About Burnout:a Systematic Review and Metasynthesis. J Gen Intern Med.

[22] Chow SK, Francis B, Ng YH, Naim N, Beh HC, Ariffin MAA (2021). Religious Coping, Depression and Anxiety among Healthcare Workers during the COVID-19 Pandemic:A Malaysian Perspective. Healthcare (Basel).

[23] Lee RLT, West S, Tang ACY, Cheng HY, Chong CYY, Chien WT (2021). A qualitative exploration of the experiences of school nurses during COVID-19 pandemic as the frontline primary health care professionals. Nurs Outlook.

[24] Houghton C, Meskell P, Delaney H, Smalle M, Glenton C, Booth A (2020). Barriers and facilitators to healthcare workers'adherence with infection prevention and control (IPC) guidelines for respiratory infectious diseases:a rapid qualitative evidence synthesis. Cochrane Database Syst Rev.

[25] Arshad MS, Hussain I, Nafees M, Majeed A, Imran I, Saeed H (2020). Assessing the Impact of COVID-19 on the Mental Health of Healthcare Workers in Three Metropolitan Cities of Pakistan. Psychol Res Behav Manag.

